# Environmental sensitivity and psychosocial characteristics in junior high school students with school refusal

**DOI:** 10.3389/fpsyt.2026.1709549

**Published:** 2026-02-24

**Authors:** Satoshi Nobusako, Harumi Mouri, Emiko Takata

**Affiliations:** 1 Neurorehabilitation Research Center, Kio University, Nara, Japan; 2 Graduate School of Health Sciences, Kio University, Nara, Japan; 3 Faculty of Education, Kio University, Nara, Japan

**Keywords:** adolescents, environmental sensitivity, highly sensitive child, interpersonal sensitivity, school refusal, stress responses

## Abstract

**Objective:**

Environmental sensitivity is a temperamental trait characterized by heightened responsiveness to environmental stimuli. Although individual differences in sensitivity have been associated with psychological adjustment, their role in school refusal (SR) remains unclear. This study aimed to investigate the environmental sensitivity of junior high school students with SR experience, as well as its relationship with stress responses and interpersonal sensitivity.

**Methods:**

Sixteen students with SR experience and seventeen students with regular attendance (RA), along with their parents, participated in the study. Students completed the Japanese version of the Highly Sensitive Child Scale for Adolescence (J-HSCS), the Children’s Stress Response scale (CSR), and the Short Forms of the Children’s Interpersonal Sensitivity Measure (CISM). Parents also completed the J-HSCS as a proxy measure of their child’s environmental sensitivity.

**Results:**

The SR group showed higher overall mean J-HSCS scores, particularly in the Low Sensory Threshold and Ease of Excitation subscales, as well as in stress responses and negative interpersonal sensitivity. Correlational analyses revealed associations among environmental sensitivity, stress responses, and interpersonal sensitivity. Moreover, there were positive correlations between student and parent ratings on several J-HSCS items, suggesting parental recognition of their child’s sensitivity regardless of SR status. Given the exploratory nature of the analyses, these findings should be interpreted cautiously.

**Conclusion:**

These findings suggest that higher environmental sensitivity may be associated with the psychological and social difficulties observed in students with SR. Individualized support strategies that acknowledge the dual nature of sensitivity—for better and for worse—may help inform prevention and intervention efforts for SR.

## Highlights

- Environmental sensitivity was greater in students with school refusal (SR).
- The SR group showed heightened reactivity to sensory and emotional environmental stimuli.
- Environmental sensitivity was associated with stress responses and interpersonal sensitivity.
- Parent and child sensitivity scores were positively correlated.
- Higher sensitivity may be associated with SR and may inform tailored support strategies.

## 1 Introduction

In recent years, school refusal (SR) has been widely recognized internationally as a serious issue that poses significant risks in academic, social, and personal domains (1). SR behavior has been associated with a decline in academic performance in the short term and with severe long-term impacts on social participation, economic independence, and physical and mental health (2). Although SR is discussed globally, its operationalization varies across countries and research traditions. For example, SR has been defined in ways that differentiate it from truancy based on features such as parental awareness of the absence and the presence of emotional distress, and estimates of how often SR occurs vary accordingly. Indeed, depending on how SR is operationalized, it has been reported to occur among approximately 0.4% to 5.4% of youth (3). In addition, some studies adopt a broader construct, SR behavior, which includes students who refuse to attend school or show persistent difficulties in remaining in class; using this broader definition, it has been suggested that up to 28% of students may express SR behavior at some point during their academic career (4). These cross-cultural perspectives underscore that reported rates and thresholds depend on the definition used, and that SR-related phenomena may manifest and be quantified somewhat differently across contexts. In Japan as well, the number of students experiencing SR has continued to rise year by year. In 2023, the number of SR cases in elementary and junior high schools reached 346,482, marking the eleventh consecutive year of increase and the highest figure on record—highlighting a deeply concerning situation. According to the definition provided by the Ministry of Education, Culture, Sports, Science and Technology in Japan, SR refers to students who do not attend school or are unable to attend school due to some psychological, emotional, physical, or social reasons or background factors, excluding cases caused by illness or economic reasons. This definition suggests that a variety of factors underlie SR.

Factors associated with SR in children include, first and foremost, mental health problems such as anxiety, depression, and stress. These negative emotional states have been reported to be strongly related to SR behavior (3, 5). In addition, difficulties in emotion regulation are considered an important intra-individual risk factor, and the development of emotional regulation skills has been identified as effective in the prevention and intervention of SR (6, 7). Furthermore, low social functioning and immature interpersonal skills have also been reported to be closely associated with SR (8), and this tendency is particularly pronounced in children with neurodevelopmental conditions such as Autism Spectrum Disorder (ASD) (9, 10). Similarly, adolescents with Attention Deficit/Hyperactivity Disorder (ADHD) also show a high risk of SR, which is reportedly associated with high levels of anxiety and negative attitudes toward school (11). In addition, bullying victimization is considered a major external factor contributing to SR (12), and anxiety symptoms such as separation anxiety and school phobia (5), as well as dysfunctional parenting styles and poor family functioning (4), have also been reported as associated factors. However, it remains unclear whether a high sensitivity to environmental stimuli—namely, a highly sensitive temperament—is involved as an internal factor related to SR.

The term Highly Sensitive Child (HSC) refers to a temperament trait characterized by heightened sensitivity to environmental stimuli and describes children who tend to respond strongly to physical and social stimuli (13, 14). This trait is understood within the framework of “Sensory Processing Sensitivity (SPS),” which includes aspects such as deep processing of sensory information, strong emotional reactivity, heightened awareness of subtle changes in the environment, and vulnerability to overstimulation (14, 15). HSC is considered a phenotypic manifestation of innate individual differences in “Environmental Sensitivity” (16). The Highly Sensitive Child Scale (HSCS), developed by Pluess et al. (13), is widely used to assess this trait, and its three-factor structure has been confirmed: Ease of Excitation (EOE), Low Sensory Threshold (LST), and Aesthetic Sensitivity (AES) (17, 18). In this study, we adopt the framework of Environmental Sensitivity (16) as a broad theoretical construct, with SPS as its core temperamental component, and use the Japanese version of Highly Sensitive Child Scale for Adolescence (J-HSCS) (19) as a validated measure of this trait.

In recent years, numerous studies have shown that children with high sensitivity traits, such as HSC, are more susceptible to stress and internalizing problems under negative environmental conditions, while they are also more likely to benefit from positive environments—a characteristic referred to as *differential susceptibility* (20–22). This heightened sensitivity is considered to have a certain genetic basis, including variants such as DRD4 and 5-HTTLPR (23, 24), and it is suggested that the expression of sensitivity is influenced by interactions with external factors such as parenting environment and life events (17, 25). Therefore, HSC is increasingly being understood not as a developmental vulnerability but rather as a form of *developmental plasticity* or an *innate individual difference* (26). However, while HSC is recognized as an important internal trait interacting with the environment, its association with children who struggle with school adjustment remains insufficiently understood.

To address this gap, it is important to consider how heightened responsivity to environmental input may shape school attendance behaviors through affective and social mechanisms. SR is often conceptualized as a heterogeneous phenomenon that can be maintained by functions such as the avoidance of school-based stimuli that elicit negative affectivity and distress, and these functions provide a useful framework for specifying where sensitivity may operate (27). In classroom settings, highly sensitive students may be more prone to sensory overstimulation (e.g., noise and other sensory stimuli) and emotional overload, which may increase distress and motivate avoidance (28). In peer contexts, heightened responsivity to interpersonal cues may amplify social-evaluative concerns, potentially increasing social anxiety and withdrawal that are commonly implicated in school refusal (29). Under academic demands, sensitivity may function as a stress-amplifying factor when coping resources are limited, but may also be buffered in supportive environments that reduce overstimulation and provide appropriate accommodations, consistent with differential susceptibility (14, 30). Articulating these pathways may help clarify why environmental sensitivity could be linked to school refusal in some adolescents and protective in others, depending on environmental conditions.

As noted above, SR has been linked to neurodevelopmental conditions such as ASD and ADHD. Importantly, SPS—the temperamental core of environmental sensitivity—has also been shown to overlap with ASD-related characteristics in non-clinical samples and trait-space analyses, with SPS subdomains (particularly EOE and LST) correlating with autistic traits (31, 32). At the same time, SPS is considered conceptually distinct from ASD despite partial phenomenological overlap in sensory responsivity, underscoring the need for careful differentiation when examining school adjustment outcomes (33). Therefore, because the present study aimed to specifically examine the association between environmental sensitivity (SPS/HSC traits) and SR, we excluded participants with clinically diagnosed neurodevelopmental disorders (e.g., ASD/ADHD) to reduce diagnostic heterogeneity and potential confounding effects, and to maintain a more homogeneous sample for isolating the contribution of environmental sensitivity to SR.

In this study, we investigated environmental sensitivity among junior high school students who were currently experiencing SR or had a past history of SR, as well as those with no such experience. Using the HSC Scale (13, 19), we assessed levels of environmental sensitivity, along with measures of stress responses and interpersonal sensitivity. We then compared these indices between students with and without SR experiences and examined the associations among them. Furthermore, in order to explore whether the extent to which parents understand their child’s environmental sensitivity is involved in the manifestation of SR behavior, we also collected parent-reported data using the HSCS. We then analyzed the relationship between the child’s self-reported HSCS scores and their parents’ reports. Given the modest sample size and the broad range of psychosocial indices examined, the present study should be considered exploratory and hypothesis-generating.

## 2 Materials and methods

### 2.1 Participants

Typically developing junior high school students and their parents participated in this study. Participants were recruited through (i) parent support groups for SR, (ii) local educational support centers, and (iii) public junior high schools. Students and their parents were provided with written information describing the purpose and procedures of the study, and those who provided informed consent participated. In total, 159 student–parent dyads were approached, of whom 33 dyads agreed to participate (participation rate: 20.8%); the remaining 126 dyads did not participate because no response was received and the families could not be reached for follow-up.

The study included two groups: (1) 16 students (mean grade ± SD = 2.25 ± 0.75; 1 male, 15 female) and their parents who were classified as having SR during junior high school, and (2) 17 students (mean grade ± SD = 1.94 ± 0.87; 5 male, 12 female) and their parents who had no SR experience and showed regular attendance (RA). Grade level was recorded according to the Japanese junior high school system, in which Grades 1, 2, and 3 correspond to international grade levels 7, 8, and 9, respectively. Among the 16 students in the SR group, 14 were currently experiencing SR, and 2 had a past history of SR during junior high school.

The inclusion criteria for the SR group followed the definition of SR used in surveys by the Ministry of Education, Culture, Sports, Science and Technology (MEXT), Japan (34): students who “do not attend school or are unable to attend despite wishing to, due to psychological, emotional, physical, or social factors/background (excluding long-term absences due to illness or economic reasons),” and who were absent from school for “30 days or more within the school year, consecutively or intermittently.” Based on this definition and criterion, only students who were jointly identified as having SR by the homeroom teacher, school nurse teacher, school counselor, and school physician, by consensus at a school case conference, were included in the SR group. All 16 students in the SR group met the above criterion of ≥30 days of absence during junior high school; however, more detailed information regarding the duration and severity of SR (beyond this criterion) was not systematically collected.

The primary aim of this study was to examine the association between SR and environmental sensitivity traits. Because both SR behaviors and environmental sensitivity traits have been linked to neurodevelopmental disorders such as ASD (31–33), participants with neurodevelopmental disorders were excluded to reduce diagnostic heterogeneity and potential confounding and to maintain a more homogeneous sample. Exclusion criteria were: (1) SR attributable to a general medical condition (e.g., illness or infectious disease such as influenza), injury (e.g., falls or traffic accidents), or physical disability (e.g., cerebral palsy, hemiplegia, or muscular dystrophy); (2) a clinical diagnosis of a neurodevelopmental disorder, including ASD, ADHD, developmental coordination disorder, or specific learning disorder; and (3) intellectual disability. Eligibility was confirmed based on the results of regular health checkups conducted by school physicians or the participant’s primary care physician, as well as interviews with parents.

All experimental procedures were approved by the local ethics committee of the institution to which the authors belong (approval number: R5-46). The study involved minimal risk to participants, and no personally identifying information was collected. The junior high school students and their parents provided background information and written informed consent. The procedures complied with the ethical standards of the 1964 Declaration of Helsinki regarding the treatment of human participants in research.

### 2.2 Procedures

Students in both groups completed the Japanese version of the Highly Sensitive Child Scale for Adolescence (J-HSCS), the Child’s Stress Response scale (CSR), and the Short Forms of the Children’s Interpersonal Sensitivity Measure (CISM). In addition, the parents of these students completed the J-HSCS to report their perceptions of their child’s environmental sensitivity.

### 2.3 Japanese version of highly sensitive child scale for adolescence

The Japanese version of the Highly Sensitive Child Scale for Adolescence (J-HSCS) is a self-report questionnaire developed for junior and senior high school students in Japan, based on the original Highly Sensitive Child Scale (HSCS) developed by Pluess et al. (13, 19). This scale aims to measure individual differences in sensitivity to environmental stimuli and consists of 11 items with a three-factor structure: Ease of Excitation (EOE; 5 items), Low Sensory Threshold (LST; 2 items), and Aesthetic Sensitivity (AES; 4 items) (13, 19). In addition to scores for each subscale (mean values), a total average score across all 11 items is calculated, with higher scores indicating a greater level of sensory processing sensitivity. Exploratory and confirmatory factor analyses supported a bifactor model including an orthogonal general factor, consistent with the structure of the original version. The scale has demonstrated good construct validity and internal consistency (Cronbach’s α: EOE = .78, LST = .71, AES = .70) (19). The J-HSCS is gaining attention not only as a tool for identifying individual vulnerability to negative stimuli but also for its ability to capture vantage sensitivity, or the potential to benefit from supportive environments (21, 35–37). In this study, the J-HSCS was used to assess adolescents’ self-reported sensory processing sensitivity.

Moreover, to evaluate environmental sensitivity from a multi-informant perspective, we also incorporated parent-reported assessments of their children’s sensitivity. This approach, based on previous research (17, 18, 38), aimed to examine the degree to which parents are aware of their children’s sensitivity traits. Specifically, in this study, parents completed the J-HSCS by responding to each item based on their perception of their child, allowing us to explore whether parental understanding of their child’s sensitivity is associated with school refusal behavior. However, it should be noted that the parent-report version of the J-HSCS has not yet been fully validated in Japan. Therefore, parent-reported J-HSCS scores in the present study should be interpreted with caution and considered exploratory.

### 2.4 Children’s stress response

The Children’s Stress Response scale (CSR) is a self-report questionnaire designed to assess children’s stress responses in a simple and efficient manner. It is intended for children from approximately 9 years of age (upper elementary school) to high school students (39, 40). The scale consists of 12 items that reflect physical, motivational, and emotional reactions to stress. Each item is rated on a 4-point Likert scale ranging from “not at all applicable (0)” to “very applicable (3),” with higher total scores indicating stronger stress responses.

The CSR comprises three subscales: Anger, which includes items such as feeling irritated, annoyed for no reason, or quick-tempered (Items 3, 7, 11); Apathy, which reflects difficulty concentrating, lack of motivation, difficulty making an effort, and a general sense of sluggishness (Items 1, 4, 8, 12); and Depression/Physical Reaction, which includes feelings of wanting to cry, being down, experiencing heart palpitations, and physical symptoms such as stomachaches and headaches (Items 2, 5, 6, 9, 10).

The total score ranges from 0 to 36, and subscale scores can also be calculated. The scale demonstrates high internal consistency, with Cronbach’s alpha coefficients reported as 0.87 for Anger, 0.82 for Apathy, and 0.73 for Depression/Physical Reaction (39). Moreover, CSR scores have shown significant positive correlations with the Public Health Research Foundation Type Stress Inventory (PSI), supporting its concurrent validity (39).

### 2.5 Short forms of the children’s interpersonal sensitivity measure

The Short Forms of the Children’s Interpersonal Sensitivity Measure (CISM) is a revised and shortened version of the interpersonal sensitivity scale originally developed by Kouda and Hidaka (41), adapted to be more accessible for children (42). This scale aims to assess the tendency to respond sensitively to others’ behaviors and attitudes. Exploratory factor analysis confirmed a two-factor structure consisting of Negative Interpersonal Sensitivity and Positive Interpersonal Sensitivity, with a total of 11 items.

Negative Interpersonal Sensitivity reflects a tendency to experience anxiety or fear in response to others’ evaluations or attitudes, while Positive Interpersonal Sensitivity represents a tendency to feel reassurance when exposed to others’ positive verbal and nonverbal cues. Higher scores indicate greater interpersonal sensitivity.

The scale has demonstrated high internal consistency, with Cronbach’s alpha coefficients of.90 for Negative Interpersonal Sensitivity and.91 for Positive Interpersonal Sensitivity. In addition, moderate correlations with related scales—such as measures of interpersonal vulnerability and empathic sensitivity—support the concurrent validity of the scale. In the present study, this short version of the CISM was used to assess children’s interpersonal sensitivity traits.

### 2.6 Statistical analysis

Based on the results of the group comparisons of J-HSCS, a *post hoc* power analysis was conducted using G*Power 3.1.9.7 (43, 44). Using the effect size, α, sample size, and number of groups, we calculated [1-β] (statistical power).

We conducted statistical comparisons of each variable measured between the SR and RA groups. For sex, we used the chi-square test. For grade level, J-HSCS variables, CSR variables, and CISM variables, we first assessed the normality of distributions using the Shapiro–Wilk test. Variables that were normally distributed were compared between groups using independent samples t-tests, whereas those that were not normally distributed were compared using the Mann–Whitney U test.

To examine the relationships among environmental sensitivity, stress responses, and interpersonal sensitivity, we conducted correlation analyses between J-HSCS variables, CSR variables, and CISM variables. Based on the results of the Shapiro–Wilk test, Pearson correlation coefficients were used for variables with normal distributions, and Spearman’s rank correlation coefficients were used for those without.

Finally, to investigate whether the extent to which parents understand their child’s environmental sensitivity is associated with SR, we performed correlation analyses between the child’s self-reported J-HSCS scores and parent-reported J-HSCS scores. Again, Pearson correlation coefficients were used for normally distributed variables, and Spearman’s rank correlation coefficients were used for non-normally distributed variables, as determined by the Shapiro–Wilk test.

We set the significance level at α = 0.05 for all analyses. Because the present study was exploratory, we did not apply a formal correction for multiple comparisons; therefore, all p-values are unadjusted and should be interpreted cautiously. In addition, we calculated the effect size. All statistical analyses were conducted using SPSS version 29.0.2.0 (IBM Corporation, Armonk, NY, USA).

## 3 Results

Using an effect size based on the group comparison results for the J-HSCS (*d* = 1.09), a sample size of 16 for the SR group and 17 for the RA group, and an α level of 0.05, a *post hoc* power analysis was conducted using G*Power 3.1.9.7. The result showed a [1-β] (power) of 0.8579382.

**Table 1** summarizes all measured data from the participants. There were no significant differences between the SR and RA groups in terms of grade level (*z* = -1.015, *p* = 0.345, *r* = -0.177) or gender (*χ²*(1) = 2.972, *p* = 0.085, *φ* = 0.300).

**Table 1:** Summary of measurement data for both groups.

Group		Grade*	Sex	J-HSCS-Self report				J-HSCS-Parent report				CSR				CISM	
				LST	EOE	AES	Total	LST	EOE	AES	Total	Anger	Apathy	D&Pr	Total	NIS	PIS
School Refusal (SR)	Mean	2.25	M, N=1 F, N=15	5.22	5.16	5.39	5.26	5.81	5.38	5.33	5.44	5.88	7.69	7.69	21.25	3.03	3.66
	SD	0.75		1.37	0.89	1.00	0.71	1.21	0.91	0.87	0.76	2.26	2.75	3.82	7.03	0.61	0.54
	Min	1		2.00	3.80	3.75	3.91	3.50	4.00	3.00	4.27	2.00	2.00	0.00	7.00	2.00	2.00
	Max	3		7.00	7.00	7.00	6.27	7.00	7.00	6.50	6.82	9.00	12.00	15.00	33.00	4.00	4.00
	Skewness	-0.444		-0.791	0.500	-0.008	-0.003	-0.536	0.272	-1.057	-0.021	0.090	-0.408	-0.015	-0.212	-0.218	-1.858
	Kurtosis	-1.062		0.043	-0.731	-1.081	-1.065	-1.102	-0.968	1.668	-1.236	-1.097	-0.734	-0.141	-0.796	-1.078	4.618
Regular Attendance (RA)	Mean	1.94	M, N=5 F, N=12	3.47	4.12	5.43	4.48	3.94	4.34	4.95	4.49	3.65	4.47	3.35	11.47	2.24	3.48
	SD	0.87		1.22	1.16	0.91	0.73	1.22	0.90	0.81	0.75	2.03	3.01	3.10	6.72	0.75	0.46
	Min	1		1	1.8	3.5	3	1.5	2.4	3.75	3.363636	0.00	0.00	0.00	0.00	1.00	2.50
	Max	3		6	6.6	6.75	6.181818	6	5.8	7	5.909091	7.00	11.00	10.00	26.00	3.57	4.00
	Skewness	0.114		-0.029	-0.150	-0.651	0.099	0.094	-0.241	0.805	0.415	-0.108	0.320	0.389	-0.088	-0.281	-0.523
	Kurtosis	-1.843		0.245	0.563	-0.055	1.071	-0.359	-0.060	1.247	-0.745	-0.459	-0.050	-0.872	-0.031	-0.742	-0.491

*Grade was recorded according to the Japanese junior high school system, where Grades 1, 2, and 3 correspond to international Grade levels 7, 8, and 9, respectively.

AES, Aesthetic Sensitivity; CISM, Short Forms of the Children’s Interpersonal Sensitivity Measure; CSR, Children’s Stress Response Test; D&Pr, Depression and Physical reaction; EOE, Ease of Excitation; F, Female; J-HSCS, Japanese version of the Highly Sensitive Child Scale for Adolescence; LST, Low Sensory Threshold; M, Male; Max, Maximum; Min, Minimum; N, Number; NIS, Negative Interpersonal Sensitivity; PIS, Positive Interpersonal Sensitivity; SD, Standard Deviation.

**Figure 1:**
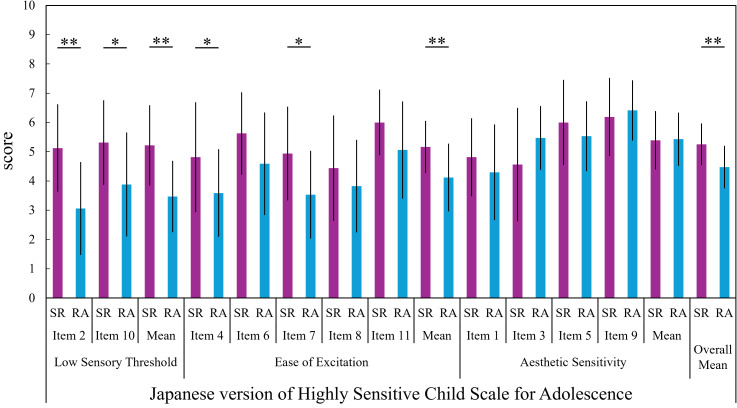
Group comparisons on the Japanese version of the highly sensitive child scale for adolescence (J-HSCS). This figure shows a comparison of scores on the Japanese version of the Highly Sensitive Child Scale for Adolescence (J-HSCS) between the School Refusal (SR) group and the Regular Attendance (RA) group. Mean scores and standard deviations for the SR group are represented by purple bars and black vertical lines, respectively; those for the RA group are represented by light blue bars and black vertical lines. Subscale interpretation. The J-HSCS assesses environmental sensitivity via three subscales: Low Sensory Threshold (sensitivity/discomfort to sensory input such as loud sounds), Ease of Excitation (being easily overwhelmed, tense, or irritated when demands are high or multiple events occur at once, including sensitivity to being observed/evaluated), and Aesthetic Sensitivity (notice of subtle environmental changes and enjoyment of positive sensory/aesthetic experiences such as pleasant smells, tastes, and music). **p < 0.01, *p < 0.05, p-values are unadjusted.

**Figure 1** presents the comparison of J-HSCS scores between the SR and RA groups. The overall mean J-HSCS score in the SR group was 5.26, whereas that in the RA group was 4.48 (see **Table 1**). The SR group showed significantly (unadjusted) higher scores than the RA group on two individual items within the LST subscale, the overall mean LST score, two items within the EOE subscale, the overall mean EOE score, and the overall mean J-HSCS score (Item 2, *t*(31) = 3.723, *p* = 0.001, *r* = 0.56, *d* = 1.34; Item 10, *z* = -2.286, *p* = 0.023, *r* = -0.40; LST, *t*(31) = 3.760, *p* = 0.001, *r* = 0.56, *d* = 1.35; Item 4, *z* = -2.084, *p* = 0.041, *r* = -0.36; Item 7, *t*(31) = 2.530, *p* = 0.017, *r* = 0.41, *d* = 0.91; EOE, *t*(31) = 2.798, *p* = 0.009, *r* = 0.45, *d* = 1.01; J-HSCS, *t*(31) = 3.023, *p* = 0.005, *r* = 0.48, *d* = 1.09). However, no significant differences were found in the individual items of the AES subscale or in the overall mean AES score.

**Figure 2:**
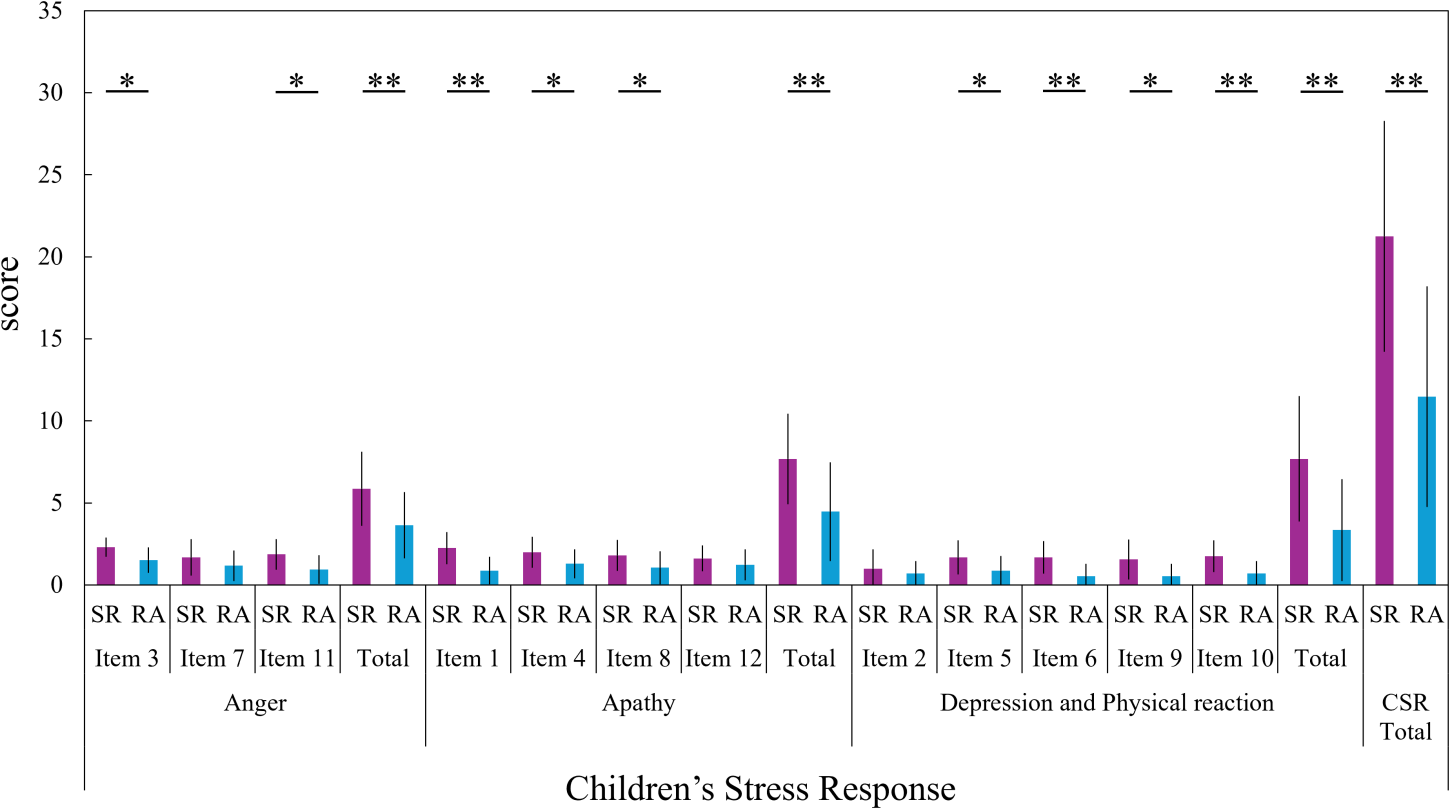
Group comparisons on the children’s stress response (CSR) scale This figure shows a comparison of Children’s Stress Response (CSR) scores between the School Refusal (SR) group and the Regular Attendance (RA) group. Mean scores and standard deviations for the SR group are represented by purple bars and black vertical lines, respectively; those for the RA group are represented by light blue bars and black vertical lines. Subscale interpretation. The CSR captures stress responses across three domains: Anger (irritability/anger reactions), Apathy (fatigue, low motivation, difficulty making an effort, and concentration problems), and Depression/Physical Reaction (depressed mood and stress-related somatic/physiological reactions such as palpitations, stomachache, headache, and feeling like crying). **p < 0.01, *p < 0.05, p-values are unadjusted.

**Figure 2** shows the comparison of CSR scores between the SR and RA groups. The SR group showed significantly (unadjusted) higher scores than the RA group on many items of the CSR as well as on the total score (Item3, *z* = -2.820, *p* = 0.010, *r* = -0.49; Item11, *z* = -2.606, *p* = 0.011, *r* = -0.45; Anger total, *t*(31) = 2.892, *p* = 0.007, *r* = 0.45, *d* = 1.01; Item1, *z* = -3.422, *p* < 0.001, *r* = -0.60; Item4, *z* = -2.051, *p* = 0.049, *r* = -0.36; Item8, *z* = -2.070, *p* = 0.045, *r* = -0.36; Apathy total, *t*(31) = 3.097, *p* = 0.004, *r* = 0.47, *d* = 1.08; Item5, *z* = -2.093, *p* = 0.045, *r* = -0.36; Item6, *z* = -3.212, *p* = 0.002, *r* = -0.56; Item9, *z* = -2.452, *p* = 0.045, *r* = -0.36; Item10, *z* = -2.887, *p* = 0.005, *r* = -0.50; Depression and Physical Reaction total, *z* = -3.036, *p* = 0.002, *r* = -0.53; CSR total, *t*(31) = 3.954, *p* < 0.001, *r* = 0.57, *d* = 1.38). Among all CSR items, only Item2, Item7, and Item12 showed no significant differences between the SR and RA groups.

**Figure 3:**
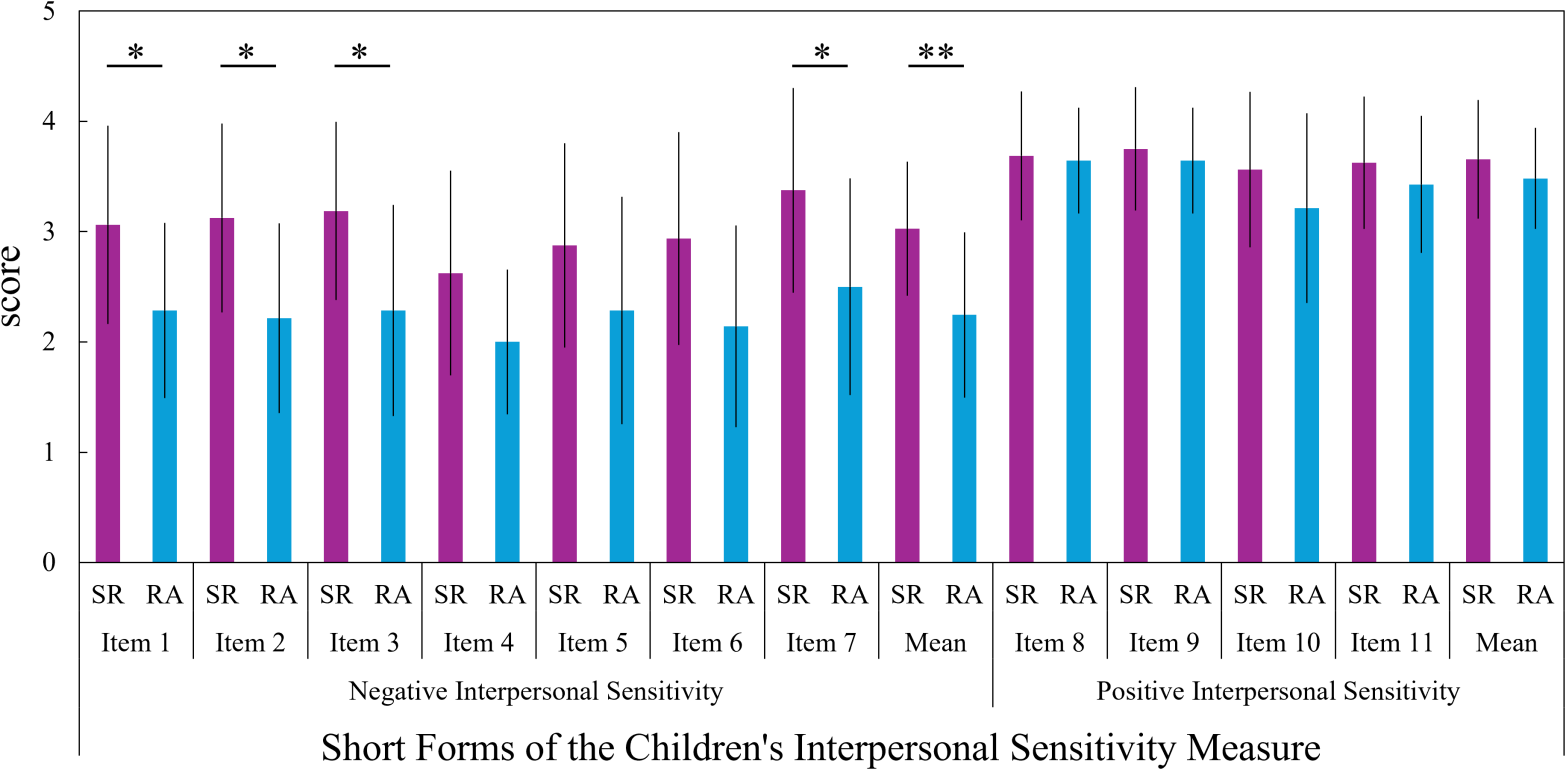
Group comparisons on the short forms of the children’s interpersonal sensitivity measure (CISM) This figure shows a comparison of scores on the Short Forms of the Children’s Interpersonal Sensitivity Measure (CISM) between the School Refusal (SR) group and the Regular Attendance (RA) group. Mean scores and standard deviations for the SR group are represented by purple bars and black vertical lines, respectively; those for the RA group are represented by light blue bars and black vertical lines. Subscale interpretation. The CISM assesses interpersonal sensitivity in two dimensions: Negative Interpersonal Sensitivity (worry/fear about others’ interest, attitudes, and evaluations—e.g., concern about being disliked or making others uncomfortable) and Positive Interpersonal Sensitivity (feeling reassured by others’ friendly and supportive verbal/nonverbal cues—e.g., kind responses and a cheerful tone of voice). **p < 0.01, *p < 0.05, p-values are unadjusted.

**Figure 3** shows the comparison of CISM scores between the SR and RA groups. The SR group showed significantly (unadjusted) higher scores than the RA group on several items within the Negative Interpersonal Sensitivity subscale of the CISM: Item 1 (*z* = -2.261, *p* = 0.031, *r* = -0.41), Item 2 (*z* = -2.458, *p* = 0.017, *r* = -0.45), Item 3 (*z* = -2.409, *p* = 0.019, *r* = -0.44), Item 7 (*z* = -2.428, *p* = 0.019, *r* = -0.44), as well as on the overall mean score for Negative Interpersonal Sensitivity (*t*(28) = 3.048, *p* = 0.005, *r* = 0.50, *d* = 1.16). However, no significant differences were found in the items or overall mean score of the Positive Interpersonal Sensitivity subscale.

**Table 2** presents the results of correlation analyses among the measured variables. The main findings from the correlation analysis conducted across the entire sample showed that the J-HSCS total mean score was significantly (unadjusted) positively correlated with all measured variables (Anger, *r* = 0.563, *p* = 0.001; Apathy, *r* = 0.468, *p* = 0.006; Depression and Physical Reaction, *r* = 0.530, *p* = 0.002; CSR total, *r* = 0.602, *p* < 0.001; Negative Interpersonal Sensitivity, *r* = 0.532, *p* = 0.003; Positive Interpersonal Sensitivity, *rs* = 0.564, *p* = 0.001). In addition, the CSR total score also showed significant (unadjusted) positive correlations with both Negative Interpersonal Sensitivity (*r* = 0.608, *p* < 0.001) and Positive Interpersonal Sensitivity (*rs* = 0.388, *p* = 0.034) in the CISM. Overall, these associations were positive and ranged from moderate to strong in magnitude, indicating that higher environmental sensitivity was accompanied by greater stress responses and interpersonal sensitivity at the correlational level. Notably, the correlations were stronger for CSR total with Negative Interpersonal Sensitivity than with Positive Interpersonal Sensitivity, suggesting a closer coupling between stress responses and negative interpersonal concerns in this sample.

**Table 2:** Correlation matrix among the measured variables.

		J-HSCS				CSR				CISM	
		LST	EOE	AES	Mean	Anger	Apathy	D&Pr	Total	NIS	PIS
J-HSCS	LST	-									
	EOE	0.500**	-								
	AES	0.119	0.039	-							
	Mean	0.721**	0.837**	0.490**	-						
CSR	Anger	0.332	0.637**	0.085	0.563**	-					
	Apathy	0.438*	0.575**	-0.134	0.468**	0.562**	-				
	D&PR	0.279	0.645**	0.038	0.530**	0.583**	0.633**	-			
	Total	0.402*	0.721**	-0.010	0.602**	0.790**	0.860**	0.900**	-		
CISM	NIS	0.346	0.653**	-0.048	0.532**	0.748**	0.316	0.566**	0.608**	-	
	PIS	0.209	0.466**	0.477**	0.564**	0.646**	0.145	0.430*	0.388*	0.488**	-

**p < 0.01, *p < 0.05, p-values are unadjusted.

AES, Aesthetic Sensitivity; CISM, Short Forms of the Children’s Interpersonal Sensitivity Measure; CSR, Children’s Stress Response Test; D&Pr, Depression and Physical reaction; EOE, Ease of Excitation; J-HSCS, Japanese version of the Highly Sensitive Child Scale for Adolescence; LST, Low Sensory Threshold; NIS, Negative Interpersonal Sensitivity; PIS, Positive Interpersonal Sensitivity.

**Table 3** presents the results of correlation analyses between children’s self-reported J-HSCS scores and parent-reported J-HSCS scores. In the SR group, significant (unadjusted) positive correlations between children’s self-reports and parent-reports were found for Item 2 (*rs* = 0.523, *p* = 0.037), LST (*rs* = 0.581, *p* = 0.018), and Item 5 (*rs* = 0.571, *p* = 0.021). In contrast, in the RA group, a significant (unadjusted) positive correlation was observed only for Item 10 (*r* = 0.604, *p* = 0.013). Across the entire sample, significant (unadjusted) positive correlations were found between children’s and parents’ J-HSCS scores for Item 2 (*rs* = 0.564, *p* = 0.001), Item 10 (*rs* = 0.535, *p* = 0.002), LST (*r* = 0.638, *p* < 0.001), Item 5 (*rs* = 0.493, *p* = 0.004), and the J-HSCS overall mean score (*r* = 0.464, *p* = 0.007).

**Table 3:** Correlation matrix between self-report and parent-report scores on the highly sensitive child scale.

	Item 2	Item 10	LST	Item 4	Item 6	Item 7	Item 8	Item 11	EOE	Item 1	Item 3	Item 5	Item 9	AES	J-HSCS
School Refusal (SR) group Parent-Child Correlation	0.523^*^	0.380	0.581^*^	0.231	-0.136	-0.054	0.141	-0.154	0.135	0.131	0.119	0.571^*^	0.048	0.235	0.462
Regular Attendance (RA) group Parent-Child Correlation	0.121	0.604^*^	0.491	-0.158	0.268	0.236	0.019	0.031	-0.106	0.316	0.396	0.287	0.098	0.311	0.108
Total Parent-Child Correlation	0.564^**^	0.535^**^	0.638^**^	0.153	0.149	0.229	0.126	0.016	0.224	0.246	0.181	0.493^**^	0.090	0.250	0.464^**^

*The numbers represent correlation coefficients. **p < 0.01, p < 0.05, p-values are unadjusted. AES, Aesthetic Sensitivity; EOE, Ease of Excitation; J-HSCS, Japanese version of the Highly Sensitive Child Scale for Adolescence; LST, Low Sensory Threshold.

## 4 Discussion

This study demonstrated that junior high school students with a history of school refusal (SR) showed significantly higher levels of environmental sensitivity, stress responses, and negative interpersonal sensitivity compared to students without such experiences. The overall mean J-HSCS score in the SR group was 5.26, whereas the RA group’s overall mean score was 4.48. According to Pluess et al. (13), the cutoff point for the HSCS is set at 4.65, with scores of 4.66 or higher indicating a Highly Sensitive Child (HSC). Based on this cutoff, it can be inferred that students in the SR group in this study exhibited a high level of environmental sensitivity. Furthermore, regardless of SR experience, significant correlations were found among environmental sensitivity, stress responses, and interpersonal sensitivity in junior high school students. In addition, there were moderate positive correlations between children’s self-reported environmental sensitivity and parent-reported sensitivity, irrespective of SR experience. Because this study employed a cross-sectional design, causality cannot be inferred; therefore, we interpret the findings in terms of associations. In addition, given the exploratory nature of the analyses and the absence of a formal correction for multiple comparisons, the results should be interpreted cautiously and viewed as hypothesis-generating.

### 4.1 Higher environmental sensitivity in the SR group

Although the J-HSCS is an instrument designed to measure SPS, we interpreted the results within the broader theoretical framework of Environmental Sensitivity (16), in line with recent research (14, 36). In this study, junior high school students with SR showed significantly higher levels of environmental sensitivity, as measured by the J-HSCS, compared to those without SR (RA group). This finding aligns with previous research on HSC, suggesting that children with heightened reactivity to environmental stimuli may be more vulnerable to psychological maladjustment under adverse conditions (13, 14, 25). In particular, the elevated scores observed in the present study on the LST (Low Sensory Threshold) and EOE (Ease of Excitation) subscales reflect a low threshold for sensory and emotional stimulation, as well as a greater susceptibility to overstimulation. These characteristics may indicate that the everyday school environment can impose excessive burdens on these students—for example, persistent classroom noise (e.g., loud voices, scraping chairs, or corridor sounds), crowded and visually busy classrooms, and frequent schedule changes or multiple simultaneous demands. In addition, interpersonal contexts may amplify distress through peer pressure, subtle teasing or exclusion, and heightened concern about social evaluation, while teacher expectations (e.g., being called on unexpectedly, strict performance demands, or constant monitoring) may further increase emotional load. Consequently, such overstimulation may be associated with school avoidance behaviors (17, 18).

While HSC traits are considered to have innate and genetic underpinnings (23, 24), their manifestation is thought to be strongly influenced by interactions with environmental factors, such as caregiving and school contexts (16, 26). The elevated sensitivity scores observed in the SR group in this study may reflect either increased exposure to inappropriate environmental stimuli or a greater vulnerability to such stimuli. In this regard, routine school demands—such as sustained sensory load (e.g., noise and crowding), peer evaluation, and teacher expectations—may be experienced as particularly taxing for students with HSC traits. Furthermore, as suggested by Iimura and Kibe (21), HSC is characterized by a “for better and for worse” nature—children with this trait tend to thrive in warm and supportive environments, while they are more likely to develop psychological difficulties in adverse or unsupportive contexts. Within this framework, it is possible that middle school students with HSC traits are more prone to SR due to heightened sensitivity to overstimulation and interpersonal stressors in school environments. Such overstimulation may accumulate as subjective psychological distress, and may be associated with difficulty attending school.

In contrast, the AES subscale did not differ significantly between groups. This pattern may be consistent with evidence that AES captures a relatively “positive” facet of environmental sensitivity (e.g., aesthetic appreciation and responsiveness to positive stimuli), whereas LST and EOE more strongly reflect susceptibility to overstimulation and negative affectivity that may be more directly relevant to school-related distress and avoidance (13, 45). In line with this distinction, AES has been linked to more adaptive functioning in some studies (e.g., better school performance and greater prosocial behavior), whereas LST/EOE show more robust associations with internalizing difficulties (46). In addition, psychometric work has suggested that AES scores can be negatively skewed (i.e., show ceiling effects) and that certain AES items may exhibit limited variability, which could reduce the likelihood of detecting between-group differences in modest samples (45). Taken together, these considerations may help explain why group differences were more evident for LST/EOE than for AES in the present sample.

In addition, our findings can be situated in relation to prior work linking SPS to ASD-related characteristics. In a non-clinical sample, Liss et al. (31) reported that the EOE and LST facets of SPS were related to autistic traits, whereas AES showed a distinct correlational pattern, suggesting that the “overstimulation/negative affectivity” facets of sensitivity may partially overlap with autism-related characteristics. Consistent with this view, trait-space analyses have further suggested that AES tends to align with more positive traits (e.g., openness), whereas EOE/LST cluster more closely with negative trait domains that include autism-related measures (32). In the present study, we excluded participants with clinically diagnosed neurodevelopmental disorders, and therefore our group differences in environmental sensitivity—most evident for LST/EOE rather than AES—are less likely to be driven by diagnosed ASD/ADHD and instead point to SPS/HSC traits as correlates of SR within a non-clinical range. At the same time, Acevedo et al. (33) emphasized that SPS is conceptually and neurobiologically distinct from ASD despite some phenomenological overlap in sensory responsivity, underscoring the importance of careful differentiation when interpreting school adjustment difficulties. Together, these comparisons help situate the present findings within existing evidence and clarify the originality of our work in positioning SR within an environmental sensitivity framework while acknowledging partial trait-level overlap with ASD-related characteristics.

### 4.2 Elevated stress responses and negative interpersonal sensitivity in the SR group

In this study, students in the SR group showed significantly higher scores on the CSR scale compared to those in the RA group. This finding is consistent with previous research indicating that many children and adolescents who experience SR exhibit stress responses such as anxiety, depression, and apathy (39, 40). These results suggest that psychological and interpersonal difficulties in school life may accumulate as sources of stress, potentially associated with SR behaviors. Furthermore, the finding that the SR group showed significantly higher scores on Negative Interpersonal Sensitivity in the CISM suggests that a heightened sensitivity to others’ behaviors and evaluations may be a common psychological trait among students with SR (41, 42). While interpersonal sensitivity can reflect rich emotionality and empathy, it also represents a vulnerability to interpersonal stress, embodying a dual-sided nature (47). In school environments, which are rich in interpersonal stimuli, this vulnerability may be associated with increased psychological burden and school refusal behavior.

### 4.3 Associations between environmental sensitivity, stress responses, and interpersonal sensitivity

Furthermore, this study found significant positive correlations between environmental sensitivity, stress responses, and interpersonal sensitivity. These results suggest that adolescents with SR experience may possess heightened reactivity to sensory and emotional stimuli, and that such high sensitivity may be linked to greater daily stress responses and emotional fluctuations in interpersonal contexts. Indeed, Pluess et al. (13) and Lionetti et al. (48) have reported that children with HSC traits are more likely to exhibit depressive and anxious tendencies in stressful environments, and that such traits are associated with heightened emotional reactivity. In addition, the development studies of the CSR have pointed out that stress responses such as anxiety and apathy play a central role in the background of SR (39, 40), and the present findings are consistent with this framework.

In addition, the correlation between HSC and CISM was particularly strong for Negative Interpersonal Sensitivity, suggesting that children with high sensitivity may be overly reactive to others’ behaviors and evaluations. This heightened sensitivity could be related to fluctuations in self-evaluation and the amplification of interpersonal stress (41, 42). Taken together, the HSC, CSR, and CISM scales appear to be closely interrelated, suggesting a pattern of associations whereby high sensitivity is associated with school adjustment and emotional stability through increased susceptibility to psychological stress and vulnerability in interpersonal relationships. On the other hand, characteristics of HSC—such as high empathy and a deep processing of information—have also been reported to serve as adaptive strengths in supportive environments (14, 21). These findings underscore the need for support strategies that acknowledge the dual nature of these internal traits.

### 4.4 Agreement between child- and parent-reported environmental sensitivity

This study also examined the correlation between children’s self-reported environmental sensitivity (J-HSCS Self-Report) and parental assessments of their children’s environmental sensitivity (J-HSCS Parent-Report). The purpose of this analysis was to explore whether the degree of parental understanding or awareness of their child’s sensitivity might be associated with the background of SR, particularly in the SR group. The results showed significant positive correlations between child and parent ratings for three items in the SR group, one item in the RA group, and five items in the total sample. A significant correlation was also found for the overall mean score of the J-HSCS in the full sample. Interestingly, the number of items showing significant correlations was greater in the SR group, and no tendency was observed indicating that parents of adolescents with SR experience had markedly poorer understanding of their children’s environmental sensitivity. One possible explanation for the relatively stronger parent–child agreement observed in the SR group is that SR may increase parents’ opportunities to observe their child’s day-to-day stress reactions and sensitivity-related behaviors, for example through more time spent at home, more frequent discussions about school-related distress, or closer monitoring of the child’s well-being. In addition, because SR is often accompanied by salient emotional and somatic complaints, parents of adolescents with SR may become more attentive to subtle signs of overstimulation or fatigue, which could enhance alignment between children’s self-reports and parental perceptions (5). From this perspective, greater agreement in the SR group may reflect heightened parental awareness of the child’s distress and sensitivity, rather than superior accuracy per se. At the same time, it is also possible that SR-related family stress, increased parental vigilance, or shared perceptions within the family context may contribute to stronger concordance. More broadly, agreement between self- and parent-reports was not uniformly high, which is consistent with prior observations that environmental sensitivity involves internal reactivity that may not be readily observable to others (13, 48). Parental ratings may also be influenced by subjective perceptions (e.g., parenting stress, parent–child relationship dynamics, or parents’ own temperamental characteristics), which could contribute to discrepancies between informants (17, 49). These findings suggest that the accuracy of parental evaluations of HSC traits and their level of understanding of their child are unlikely to be major contributing factors to SR.

On the other hand, Pluess et al. (13) and Lionetti et al. (48) have noted that environmental sensitivity is an internally driven temperamental trait that includes high internal reactivity, which is often difficult to observe externally. Therefore, there may be inherent limitations in the degree of agreement between self-reports and other-reports. Moreover, parental assessments may be influenced by subjective perceptions shaped by parent–child relationships, parenting stress, or the parents’ own temperamental traits (17, 49), suggesting that there may be individual differences in the accuracy with which HSC traits are observed and evaluated by caregivers.

Nevertheless, the overall finding of significant positive correlations between child and parent ratings supports the notion that using the J-HSCS as a parent-report measure may have acceptable reliability and clinical utility (21). Future research should explore how parents’ awareness of their children’s environmental sensitivity influences their interactions and support styles, and further examine how such awareness may contribute to the prevention and intervention of SR.

### 4.5 Implications for prevention and intervention

Importantly, environmental sensitivity should not be conceptualized as inherently maladaptive; rather, consistent with the differential susceptibility perspective, it may confer vulnerability in adverse contexts while supporting better adjustment in supportive environments. Given the cross-sectional design, we interpret our findings as associations, and we emphasize that future work incorporating measures of environmental quality is needed to test sensitivity × environment interaction effects.

The present findings suggest several practical implications for prevention and intervention in school settings. First, because students with SR showed elevated LST/EOE and stronger links between sensitivity and stress responses, reducing sensory and emotional overload in everyday school environments may be beneficial. Reasonable accommodations could include providing predictable routines, allowing access to quieter spaces, minimizing unnecessary noise or crowding when feasible, and offering flexibility in task demands and pacing during periods of heightened distress, consistent with the “for better and for worse” perspective on environmental sensitivity (13, 21). Second, given the associations between environmental sensitivity and stress responses, interventions that strengthen emotion regulation and stress-management skills (e.g., identifying early signs of overload, breathing/grounding strategies, and graded exposure to challenging contexts) may help students cope with school-related demands (13). Third, the robust association with Negative Interpersonal Sensitivity highlights the potential value of addressing threat-focused social interpretations and interpersonal anxiety, for example through cognitive-behavioral strategies that target maladaptive appraisal and avoidance in peer-evaluative situations (31). Finally, because child–parent ratings of sensitivity showed significant agreement at the overall level, collaborative psychoeducation for caregivers and school staff may support earlier recognition of sensitivity-related overload and facilitate individualized support plans that balance protection from overstimulation with gradual re-engagement (13).

### 4.6 Limitations of the current study and future directions

This study has several limitations that should be considered when interpreting the findings. First, the sample size was modest (SR = 16; RA = 17), and participation was constrained by recruitment yield. Although 159 student–parent dyads were approached, only 33 dyads participated (participation rate: 20.8%), and additional recruitment was not feasible because the remaining dyads did not respond and could not be reached for follow-up. Therefore, the final sample size was determined pragmatically rather than by an *a priori* power calculation. While a *post-hoc* power analysis using G*Power indicated power above the conventional threshold (1−β = 0.8579), such estimates should be interpreted cautiously and do not substitute for prospective sample size planning. Moreover, the SR group was predominantly female (15/16), which limits generalizability and precludes examination of gender-specific patterns. Accordingly, future studies should employ prospectively planned sample sizes based on *a priori* power analyses and recruit larger, more diverse, and gender-balanced cohorts to improve statistical precision, generalizability, and the robustness of conclusions regarding the association between environmental sensitivity and SR.

Second, because this study used a cross-sectional design, causality cannot be inferred, and the directionality of associations between environmental sensitivity and SR remains unclear. Longitudinal designs are needed to clarify temporal pathways and examine whether environmental sensitivity predicts the onset, persistence, or remission of SR tendencies over time.

Third, the present study did not examine SR subtypes (e.g., anxiety-based, avoidance-based, parent-dependent), which may have different psychosocial profiles and mechanisms. Future research should incorporate subtype classification and consider heterogeneity within SR to better tailor prevention and intervention strategies.

Fourth, although we assessed environmental sensitivity using both self-report and parent-report formats of the J-HSCS, the psychometric validity of the Japanese parent-report version has not yet been sufficiently established. In addition, reliance primarily on self- and parent-reports may introduce informant-related biases. Future studies should further validate the parent-report measure and adopt multi-informant approaches (e.g., teacher reports and/or behavioral or observational indicators) to triangulate sensitivity, stress, and interpersonal functioning.

Fifth, to isolate the association between environmental sensitivity and SR, we excluded participants with clinically diagnosed neurodevelopmental disorders. While this approach reduces diagnostic heterogeneity and potential confounding, it limits applicability to real-world SR populations, where comorbidity with ASD/ADHD is common. Future work should extend these analyses to SR samples that include neurodevelopmental comorbidities to enhance ecological validity.

Sixth, the SR group included both students who were currently experiencing SR and those with a past history of SR during junior high school. This composition may have introduced within-group heterogeneity, as current and past SR experiences may differ in severity, recency, and associated psychological burden. Although the subgroup with past SR was small (2 of 16), future studies should distinguish more clearly between current and remitted SR, or examine these subgroups separately, to better capture potential differences in psychosocial profiles.

Seventh, because we conducted multiple statistical tests across several questionnaire subscales and items, the probability of false-positive findings may be increased. Accordingly, given the exploratory design and modest sample size, we did not apply a formal correction for multiple comparisons; thus, results should be interpreted cautiously and viewed as hypothesis-generating. Future confirmatory studies with larger samples should pre-register primary outcomes and apply appropriate multiplicity control (e.g., Bonferroni correction or false discovery rate procedures).

Finally, the study was conducted in Japan, and definitions, thresholds, and school contexts related to SR vary across cultures. Replication in other cultural settings and cross-cultural comparative studies are therefore needed to evaluate the generalizability of the present findings.

## 5 Conclusion

This study demonstrated that junior high school students with SR experience showed significantly higher scores in environmental sensitivity—particularly in LST and EOE—as well as elevated stress responses and negative interpersonal sensitivity, compared to their peers without SR experience. Significant correlations were also found between environmental sensitivity and both stress responses and interpersonal sensitivity, suggesting that these traits are closely linked to psychological adjustment.

Furthermore, consistent correlations were observed between children’s self-reported environmental sensitivity and their parents’ assessments, regardless of SR status. This finding suggests that parental understanding of their child’s sensitivity may not be a primary factor contributing to SR.

Overall, the present findings indicate that high environmental sensitivity as a temperamental trait may be closely associated with school adjustment and the tendency toward SR. These results underscore the importance of understanding and supporting highly sensitive children in efforts to prevent and intervene in SR. In particular, individualized approaches that take into account the dual nature of sensitivity—*for better and for worse*—may be essential in both educational and clinical settings.

## Data Availability

The raw data supporting the conclusions of this article will be made available by the authors, without undue reservation.
